# Microbiological Evaluation of Thermoplastic PETG Dental Appliances Related to Surface Characteristics

**DOI:** 10.3390/polym16162354

**Published:** 2024-08-20

**Authors:** Liliana Porojan, Flavia Roxana Bejan, Emil Tirziu, Cristina Mirabela Gașpar, Alex Cristian Moza, Mihaela Ionela Gherban, Roxana Diana Vasiliu, Anamaria Matichescu

**Affiliations:** 1 Department of Dental Prostheses Technology (Dental Technology), Center for Advanced Technologies in Dental Prosthodontics, Faculty of Dental Medicine, “Victor Babeș” University of Medicine and Pharmacy Timișoara, Eftimie Murgu Sq. No. 2, 300041 Timișoara, Romania; flavia.toma@umft.ro (F.R.B.); roxana.vasiliu@umft.ro (R.D.V.); 2 Department of Animal Production and Veterinary Public Health, Faculty of Veterinary Medicine Timișoara, University of Life Sciences “King Mihai I” from Romania, Calea Aradului 119, 300645 Timișoara, Romania; emiltarziu@yahoo.com (E.T.); gasparcristina99@yahoo.com (C.M.G.); mv.alexmoza@gmail.com (A.C.M.); 3 National Institute for Research and Development in Electrochemistry and Condensed Matter, 300569 Timișoara, Romania; mihaelabirdeanu@gmail.com; 4 Department of Preventive, Community Dentistry and Oral Health, Center for Advanced Technologies in Dental Prosthodontics, Faculty of Dental Medicine, “Victor Babeș” University of Medicine and Pharmacy Timișoara, Eftimie Murgu Sq. No. 2, 300041 Timișoara, Romania; matichescu.anamaria@umft.ro

**Keywords:** dental thermoplastic PETG, Candida albicans biofilm, artificial ageing, roughness at different scale lengths

## Abstract

(1) Background: The adhesion and microbiological behaviour of thermoplastic PETG dental appliance surfaces is governed by roughness parameters. The aim of this research was to evaluate the antibiofilm activity of alkaline peroxide-based disinfectant in Candida albicans biofilms on thermoplastic PETG, related to artificial ageing and surface characteristics, on multiscale levels. (2) Methods: In the present study, two PETG materials were investigated: Crystal^®^ (Bio Art Dental Equipment, Sao Carlos, Brazil), noted as C, and Duran^®^ (Scheu-Dental GmbH, Iserlohn, Germany)—noted as D. Half of the specimens were thermally cycled (TC), resulting in four sample groups, as follows: C, CTC, D, and DTC. Surface roughness was evaluated on different scale topographies. The biofilms were grown on the surfaces. An alkaline peroxide-based disinfectant was used. Statistical analyses were performed. (3) Results: Related to nanoroughness, there are insignificant differences among materials or related to thermocycling. More irregular surfaces are associated with larger grain sizes. After thermocycling, micro-roughness values increase. Disinfectant activity decreases the amount of biofilm developed on the surfaces, significantly in the two groups, but is not correlated to the material and artificial ageing. (4) Conclusion: The impact of surface roughness (Ra) on biofilm constitution is controlled by different scale topographies.

## 1. Introduction

Thermoplastic appliance surfaces are prone to the colonisation of microorganisms in the oral environment. These stick to the surfaces of the materials through distinctive interactions. Generally, a great variety of organic molecules cover surfaces, and microorganisms are introduced through physicochemical interaction. The connection between the thermoplastic material and the microorganism may induce bond strengthening, immediately after the initial contact [1,2].

PETG is a thermoplastic material resulting from the polycondensation reactions of EG (ethylene glycol), TPA (terephthalic acid), and CHDM. PETG has an irregular molecular chemistry, which induces its amorphous features, chemical resistance, and transparency. The synthesized thermoplastic material (PETG) can be machined by pressure or injection moulding, thermoforming, or three-dimensional printing. Due to its biocompatibility and durability, the material is suitable for use in dental practice [3]. It has been observed that PETG materials retain their form in the oral environment. The mechanical characteristics of PETG are highly valued when these thermoplastics are considered for medical applications. PETG is an inexpensive and effective biomaterial. Both materials used in the study are PETGs, processed by thermoforming. The process of biofilm constitution is elaborate and multifactorial, being affected by the interaction between microorganisms and the conditions of the oral environment [4,5,6,7].

Candida albicans is a resident component of the oral cavity microbiota; generally, it does not cause any disease, but it is a fungus that may induce an infection, if the equilibrium of the oral environment is affected or disturbed. It has an intrinsic capacity to naturally attach to thermoplastics, leading to candidosis [4].

Studies have shown that surface roughness influences the formation and development of the biofilm and has a relevant function in the primer stadium of Candida albicans adhesion [8].

Understanding the degree of surface roughness and the surface topography at different scale lengths is of great interest in the scientific field. The adhesion and microbiological behaviours are governed by roughness parameters [9]. Different properties could be controlled by different scale topographies [10,11].

Chemical cleaning procedures have the advantage of being easily obtained and practised by patients, without difficulty. Effective sanitising solutions for disinfecting thermoplastics are promoted and are already well known. Corega tabs are widely marketed disinfectants and provide effective mechanical cleaning through the antimicrobial activity of alkaline peroxide, more exactly through oxygen emission. Their advantages are the absence of taste and odour, compared to other disinfectants [12,13]. Cleaning methods, as well as ageing, may change surface characteristics, with an impact on microorganism colonisation.

The objective of this study was to evaluate the antibiofilm activity of alkaline peroxide-based disinfectant in Candida albicans biofilms on thermoplastic PETG appliances related to artificial ageing and surface characteristics, on multiscale levels. The null hypotheses were as follows: 1. the type of material and artificial ageing influence the surface roughness, recorded at both micron- and nano-scales; 2. the amount of biofilm developed on the surfaces is correlated to the material, artificial ageing by thermocycling, and disinfectant activity; and 3. the impact of surface texture and roughness on biofilm constitution is controlled by different scale topographies.

## 2. Materials and Methods

### 2.1. Specimen Preparation

Two similar PETG commercial products, for dental appliances, corresponding to two different brands, were chosen for this research: Crystal^®^ (Bio Art Dental Equipment, Sao Carlos, Brazil), noted as C, and Duran^®^ (Scheu-Dental GmbH, Iserlohn, Germany)—D.

The specimens underwent the following processing steps: The sheets of PETG material were heated (30 s, 220 °C) and then vacuum-pressed (4.7 bar) above a gypsum die with flat surfaces (10 mm × 30 mm) oriented towards each other at an angle of 45°. The gypsum mols was isolated with Isofolan. The material cooled in 60 s. Models were designed in order to imitate the position of natural teeth and their average size. The thermoformed sheets were removed, and the samples were sectioned into sheets with identical sides (10 × 10 mm) and then used for analyses. The definitive thickness was variable, according to the effect of thermoforming on each material (0.75 mm for [C] and 0.68 mm for [D]).

The sample size was represented by 90 pieces of square PETG slides made out of material [C] (10 × 10 mm; a thickness of 0.75 mm), and another 90 slides made out of material [D] (10 × 10 mm; a thickness of 0.68 mm). Out of each group, half of the slides (45) were thermally cycled (TC), thus obtaining four sample groups, as follows: C, CTC, D, and DTC. 

Groups C and D were deposited in airtight glass containers with distilled water at 37°C (inside an incubator)—for two weeks. And for specimens from the CTC and DTC groups, the ageing procedure by thermocycling was applied. A total of 833 cycles were performed in two water baths cold↔warm (5↔55°C) with 30 s of dwell time (+10 s of transfer time), to imitate 30 days of intraoral utilisation [14,15]. Water thermocycling was chosen because the literature data indicated that it induces a deterioration of the PETG materials. The selected interval corresponds to the maximum use of appliances for orthodontic purposes.

### 2.2. Nanosurface Topographic Characterisation

Specimens from each set were randomly selected and assessed in a non-contact mode, with a Nanosurf Easy Scan 2 Advanced Research atomic force microscope (NanosurfAG, Liestal, Switzerland), and the average nanoroughness—Sa (nm), the maximum amplitude of heights—Sy (nm), and grain size—GS (nm) were recorded. Atomic force microscopy (AFM) provided appropriate 3D profile images and colour representations of specimen areas (2.22 μm × 2.22 μm and 4.52 μm × 4.52 μm). The recordings were performed on samples from each set. Multiple levels were taken into consideration, even at the nanoscale, in order to assess their influence on the antibiofilm activity.

### 2.3. Surface Micro-Roughness Measurements

The evaluation of the surface micro-roughness was performed with a contact profilometer (Surftest SJ 201-Mitutoyo, Kawasaki, Japan) equipped with a diamond stylus (2 μm). The measurements were made on the sample surface, in three randomly chosen areas, and then the average values were calculated. The investigation was performed on a sampling length of 0.8 mm, applying a force of 0.7 mN, and the arithmetic mean roughness (Ra) and maximum absolute vertical roughness (Rz) were achieved.

### 2.4. In Vitro Biofilm Formation Protocol 

Candida is present in the largest amount and most commonly isolated in the oral mycobiome and therefore was chosen for the study. Many Candida albicans organisms are capable of creating biofilms on abiotic areas (different materials, prostheses, and orthodontic appliances) and/or on biotic areas (teeth and mucosal cells), constituting polymicrobial communities [16]. 

The biofilms were grown on the surfaces of slides made out of PETG materials. A standard strain of Candida albicans ATCC 10231™ (Culti-Loops™ Thermo Scientific™, Waltham, MA, USA) was used. The activation of the Candida albicans strain was achieved at 37 °C/48 h on Sabouraud Dextrose Agar (Thermo Scientific™ Oxoid™, Waltham, MA, USA). Afterwards, colonies were loop-transferred in physiological saline (0.9% NaCl), and mixed and adapted to obtain a turbidity of 0.5 McFarland (0.08–0.12 OD at 625 nm). From this solution, 1 mL was added into 149 mL of Brain Heart Infusion (BHI) broth, thus obtaining the yeast suspension. The PETG slides were inserted horizontally at the bottom of 24-well flat-bottom culture plates. In each well, we added 900 μl of BHI broth and 100 μl of yeast inoculum. The wells representing the negative controls were only filled with 1000 μl of sterile BHI broth.

The culture plates were stored in the incubator (37 °C) for 48 h. After that, samples were individually rinsed in a tray of water by submerging them three times and transferred onto a new culture plate; thus, only the biofilm that formed on the slides was studied. Heat fixation of the biofilm was performed in an electric oven at 60 °C, for 20 min. During each step, the slides were handled with the dorsal side up.

The freshly prepared solution from Corega Cleanser Tablets (Stafford-Miller, Dungarvan, Ireland) was added to the wells of new culture plates, in which slides from both the contaminated and sterile lot were already transferred. As recommended by the producer, the PETG samples were removed after three to five minutes of treatment, rinsed in a tray of water, and transferred to another 24-well culture plate.

**Figure 1:**
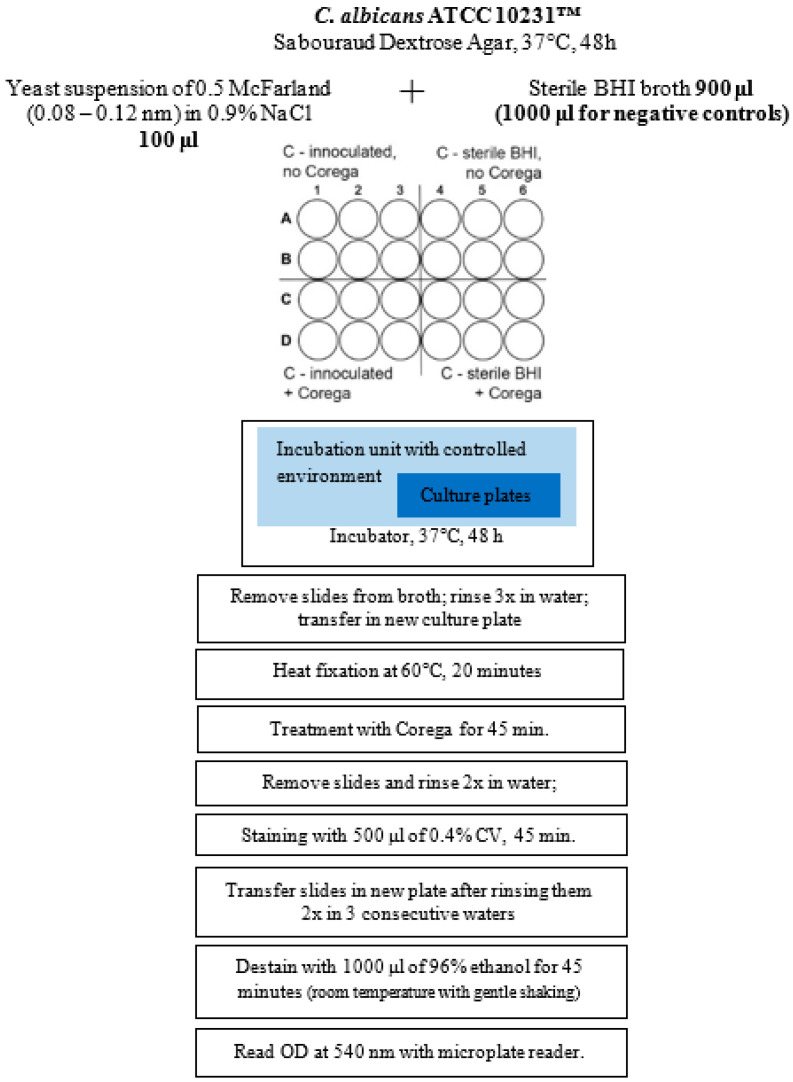
A representation of the CV microplate assay for material C.

Staining was performed for 45 min by adding to each well 500 μl of 0.4% CV (Crystal Violet solution), followed by rinsing the slides by immersing them twice in three consecutive water baths. Destaining was achieved on a clean microplate, with 1000 μl of 96% ethanol for 45 min at room temperature, with gentle shaking, and, finally, the OD—the optical density of the CV, which was bound to the adhered biofilms—was determined at 540 nm with the Tecan^®^ microplate reader—Figure 1.

### 2.5. Statistical Tests

Significance tests were effectuated using IBM SPSS Statistics software (IBM, New York, NY, USA). Means, standard deviations (SDs), and dissimilarities among variables were computed. The (unpaired) Student’s *t*-test was performed to compare two different groups (the two materials undergoing the same handling in one phase), and the Student’s (paired) *t*-test was applied to compare the results between two stages (before and after thermocycling). Pearson correlation was applied to evaluate relationships among different variables (grain size, Sa, Sy, Ra, and Rz). 

Welch’s *t*-test (Two-Sample *t*-test) and the Mann–Whitney U test were used to calculate and analyse the variations in the OD values, obtained after growing the Candida albicans on the tested PETG materials and after treating the formed biofilms with the Corega product. α = 0.05 was set as the significance level. 

## 3. Results

**Figure 2:**
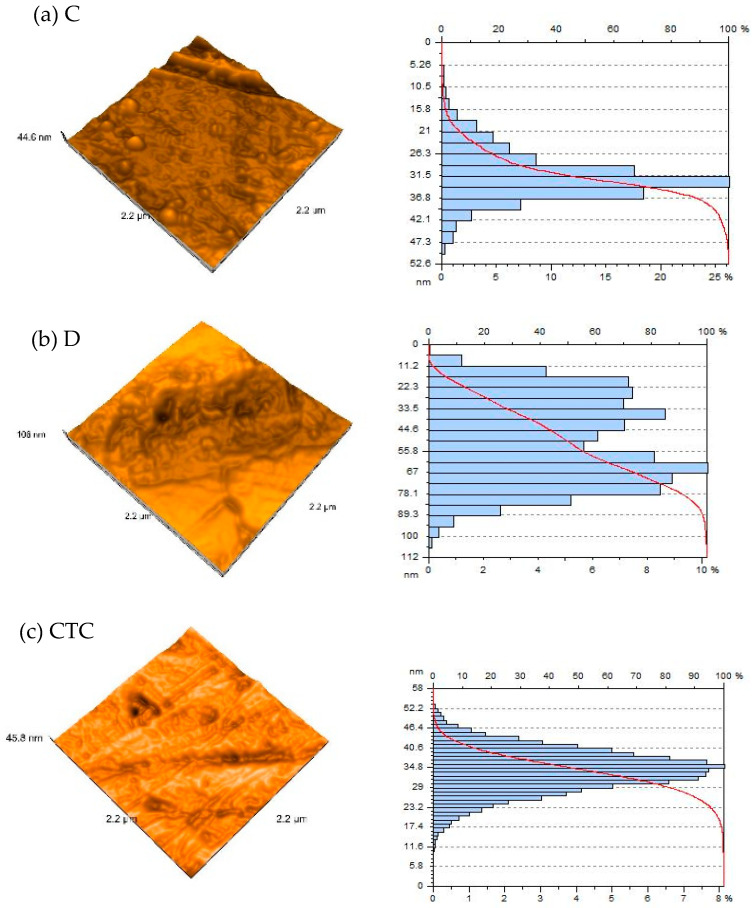

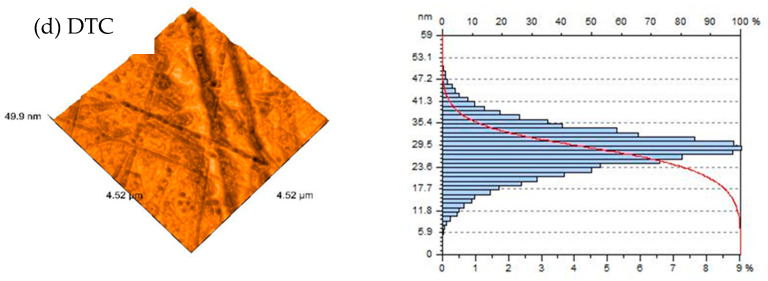
An AFM 3D representation of the surface topography and distribution of the grain sizes for each group of materials (a square area with a side of 2.22 μm was investigated): (**a**) C, (**b**) D, (**c**) CTC, (**d**) DTC.

**Figure 3:**
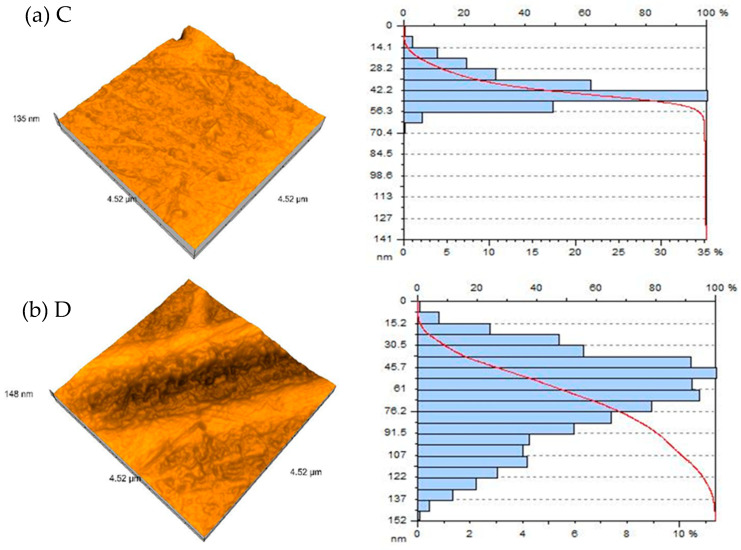

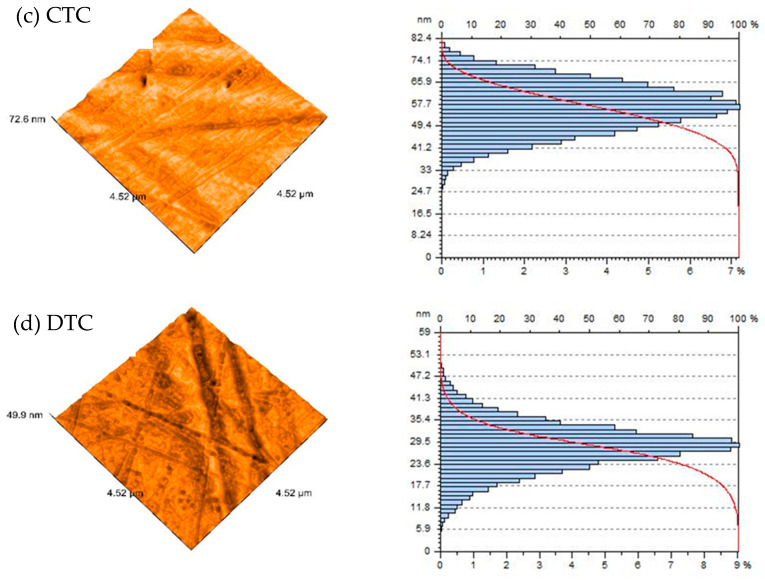
An AFM 3D representation of the surface topography and distribution of the grain sizes for each group of materials (investigated area: 4.52 μm × 4.52 μm): (**a**) C, (**b**) D, (**c**) CTC, (**d**) DTC.

High-resolution 3D AFM images reveal data scattered throughout the evaluated specimen with areas of 2.22 × 2.22 μm and 4.52 × 4.52 μm. The distribution of the grain size was assessed for both materials used in the study, for each group of samples (Figure 2 and Figure 3 and Table 1 and Table 2). More irregular surfaces are associated with larger grain sizes.

**Figure 4:**
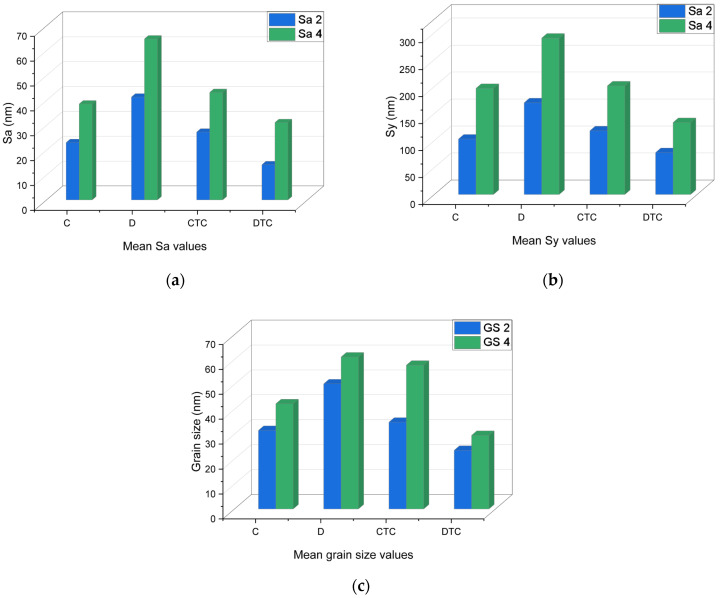
A graphical representation of the mean Sa, Sy, and grain size values. Related to the grain size (D-DTC), *p* = 0.026: (**a**) mean Sa values, (**b**) mean Sy values, (**c**) mean grain size values.

Mean values are graphically represented (Figure 4), and statistical analyses indicate that regarding nanoroughness, there were insignificant differences even among PETG materials, or related to thermocycling. For C, the values increased, and for D, they decreased after thermocycling, but not significantly. Related to the grain size, the behaviour is similar to nanoroughness, but the decrease for D, after thermocycling, is significant (*p* = 0.026). 

Thus, the highest nanoroughness and grain size values were registered for the D and CTC groups.

Calculated Pearson coefficients indicated a very strong positive correlation between Sa 2 and Sa 4 (r = 0.98), Sy 2 and Sy 4 (r = 0.98), GS 2 and GS 4 (r = 0.87).

**Figure 5:**
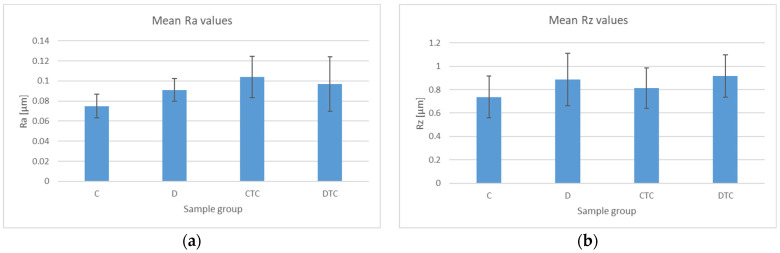
A graphical representation of the average Ra and Rz values. Related to Ra, for C-D, *p* = 0.004, and for C-CTC, *p* = 0.0007: (**a**) mean Ra values, (**b**) mean Rz values.

Roughness value measurements on a micron scale are represented graphically in Figure 5. After thermocycling, Ra and Rz values increased, significantly only for C (*p* = 0.0007). Related to the materials, significant differences were present before thermocycling (*p* = 0.004). After thermocycling, micro-roughness values were not significantly different. C was more affected by thermocycling. 

The roughness values on a nanolevel (Sa 2 and Sa 4) are strongly positively correlated (r = 0.98), but not correlated with the micro-roughness.

The first null hypothesis stated, that the category of material and artificial ageing influences the surface roughness, recorded at both micron- and nano-scales, is partially accepted. On micron-scales, roughness increases after thermocycling, significantly for C, beginning from significantly different values and becoming insignificantly different. Related to nanoroughness, material type and thermocycling have no significant influence. For C, the values increase, and for D, they decrease after thermocycling, but not significantly.

After the centralisation and analysis of the results related to biofilm formation, in the case of materials C and DTC, the number of samples was only sufficient for applying the Mann–Whitney U statistical analysis, while for CTC and D, the Two-Sample *t*-test could be applied.

The mean OD values suggest that a reduction in biofilm quantity was well observed after the disinfection with Corega in the case of all materials shown in Table 3. 

Comparing the biofilms developed on materials C (new) and CTC (thermally cycled), we found that the difference in the biofilm quantity developed in material CTC has no statistically significant difference towards material C, with *p* = 0.44 (*p* > 0.05). Similarly, insignificant differences in the amount of biofilm formation were found between materials D (new) and DTC (thermally cycled), with *p* = 0.40 (*p* > 0.05). 

**Figure 6:**
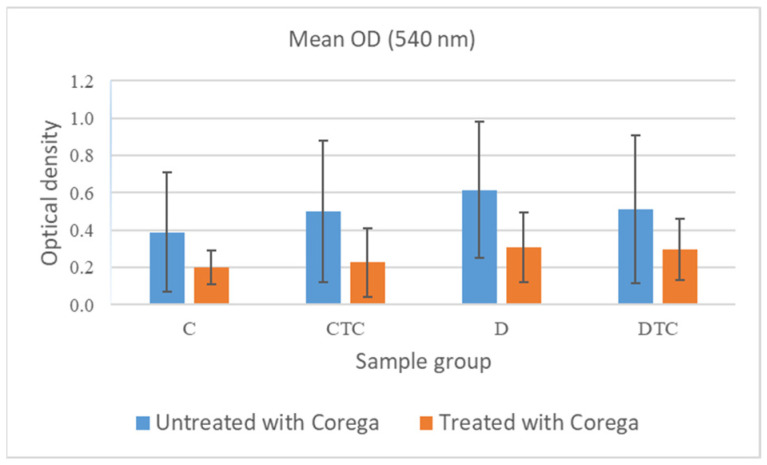
C. albicans biofilm on slides untreated and treated with Corega. For CTC and D, *p* < 0.05.

The antibiofilm action of Corega was found to be significant for the biofilm developed on materials CTC (*p* < 0.05) and D (*p* < 0.05). On the other hand, the antibiofilm action observed on the biofilms grown on materials C and DTC was not considered statistically significant, with *p* values > 0.05 (C *p* = 0.27; DTC *p* = 0.17)—Figure 6. Between materials C and CTC (*p* = 0.54) and D and DTC (*p* = 0.72), there were insignificant differences regarding the quantity of biofilm that was removed after the treatment with Corega.

According to the results obtained, insignificant correlations were observed related to the origin of the PETG materials (Brazil or Germany) and the amount of biofilm developed on their surfaces. Thus, the probability (*p*)-value was higher than α = 0.05 (Mann–Whitney U test) when comparing materials C and D, as well as CTC and DTC, both treated and untreated with Corega.

The second null hypothesis, that the amount of biofilm developed on the surfaces is correlated to the material, artificial ageing by thermocycling, and disinfectant activity, is partially accepted. Disinfectant activity decreases the amount of biofilm developed on the surfaces, significantly in the two groups, but is not correlated to the material and artificial ageing. 

Pearson correlation coefficient values show strong positive correlations between Sa and the amount of biofilm on untreated surfaces, moderate to strong between GS and the amount of biofilm on untreated surfaces, and very strong and strong positive correlations between Ra and the amount of biofilm on both untreated and treated surfaces (Table 4). 

Thus, the third null hypothesis, that the impact of surface roughness on biofilm constitution is controlled by different scale topographies, is accepted.

## 4. Discussion

Previous studies demonstrated that surface roughness, evaluated by AFM, and surface characteristics, can have an important favourable influence in the initial phases of bacterial retention to the material [1].

To explain the specific virulence of oral microorganisms, it is essential to understand the stages of biofilm constitution. The activity begins when the appliances come into contact with the intraoral medium. Macromolecules are adsorbed on their surfaces, leading to the formation of the acquired film. Thus, microorganisms become more adherent to the surface, showing active growth [17,18].

Candida albicans is known to have high virulence coefficients and the ability to adhere to surfaces of resin materials. These aspects may justify why the sanitising factors can have lower effects on the biofilm of Candida albicans, because it is able to produce high-tolerance biofilms [19].

Candida albicans is an important opportunistic pathogenic fungus because after adhesion, it generates and forms biofilms, and secretes a toxin and proteinases. It may become a pathogen agent both locally and systemically. The relevance of preserving and using removable Candida-free orthodontic appliances has to be highlighted. Therefore, they have to be cleaned and disinfected adequately, without altering their surface properties [20].

Wearing removable appliances may change the oral ecosystem, leading to an increase in the potential for the appearance of intraoral affections, like caries, halitosis, and periodontal diseases. Hence, proper sanitation and biofilm control are essential [21,22,23].

The Candida albicans biomass accumulation was indicated by a Crystal Violet assay. CV testing is the most frequently used quantitative method for identifying biomass accumulation in microplate procedures [24]. Other representative methods to describe the antifungal activity are live/dead staining images of the biofilm formed on the material surfaces, SEM analyses, and the ROS detection of intracellular reactive oxygen species development levels by fluorescent probes, and these methods should be taken into consideration for further studies.

After execution, the surface morphology of the thermoplastic appliances is smooth and uniform. But, after intraoral use, critical modifications were found, including micro-fissures and fractures all over the appliance. Analysing from a microbiological perspective, these results validate the existence of new niches for microbial multiplication and, consequently, adequate disinfection is important to hinder the propagation of microorganisms and the building of biofilms [25].

A rough surface texture has been shown to lead to predictable microbial adhesion. The rough surfaces of oral thermoplastics are associated with greater microbial accumulation [26].

The consequence of surface roughness, at both micron- and nano-scales, and particle size on cell adhesion, is debatable in the literature [10].

The ageing process, as well as the long-term use of sanitising and disinfecting products, causes an increase in surface roughness, negatively affecting microbial adhesion [27,28]. Roughness, defects, and surface appearance are key factors that induce biofilm formation [29]. It has been observed that commercially available chemicals used for cleaning dentures, such as liquid products or effervescent tablets, do not completely remove the biofilm coating, leaving viable microorganisms on the roughest or adherent surfaces [30,31]. 

Thermoplastic orthodontic removable appliances should be used for 1–4 weeks during the treatment, ensuring they are in direct contact with dental and periodontal tissues, as well as intraoral fluids, for this period. Thermoplastics might be altered by changes in the oral environment and disposal molecules, which could be harmful. In the scientific literature, topics related to the cytotoxicity of thermoplastics and clear aligners have not been sufficiently debated. Under experimental circumstances, all thermoplastics demonstrated a slight cytotoxic impact with different cell viability stages. These outcomes related to cytotoxicity level were identical to or lower than those obtained by many other orthodontic dental plastics. Intraoral use of aligners requires systematic cleaning and disinfection. Since the materials have demonstrated a slight cytotoxicity level, their clinical indication and application in the dentistry field can be considered safe [32].

The limitations of this research include the in vitro experiment, due to the lack of real conditions existing in the oral environment. The intraoral environment cannot be completely simulated with any kind of in vitro methods. Also, Candida albicans adherence was evaluated independently, but the oral microbiome interacts in complex biofilms [33]. Extensive studies should investigate the surfaces before and after various biofilm formation and disinfection protocols. 

Surface properties of materials are governed by certain ranges of size scales. Therefore, some of the PETG appliances’ key properties could be attributed to the surface scale size. For example, it was found that stiffness is controlled by large-scale topography and friction by the very smallest-scale topography. The literature data mention that surface roughness on different scales and in different grain sizes is inconclusive for cell adhesion [10]. Therefore, it is important to find out which properties are controlled by topography at which scales and to describe applications for which the multiscale roughness is important. Surface modification techniques could also represent a topic of interest.

## 5. Conclusions

1. On micron scales, roughness increases after thermocycling, significantly for C.
2. Related to nanoroughness, material type and thermocycling have no significant influence on the formation of microfilm.
3. Disinfectant activity decreases the amount of biofilm developed on the surfaces, significantly in two groups (D and CTC), but is not correlated to the material and artificial ageing.
4. The impact of surface roughness on biofilm constitution is controlled by different scale topographies.

## Figures and Tables

**Table 1:** Mean values after AFM analyses for samples with areas of 2.22 × 2.22 μm.

Group	Grain Size GS2 (nm)	Sa 2 Value (nm)	Sy 2 Value (nm)
C	31.5	23.05	102.75
D	50.2	38.58	170.11
CTC	34.8	27.27	118.13
DTC	23.5	14.55	77.72

**Table 2:** Mean values after AFM analyses for samples with areas of 4.52 × 4.52 μm.

Group	Grain Size GS 4 (nm)	Sa 4 Value (nm)	Sy 4 Value (nm)
C	42.2	41.35	196.74
D	61	64.73	289.94
CTC	57.7	43.22	201.39
DTC	29.5	31.10	133.48

**Table 3:** The biofilm average values and SD (standard deviation) OD values for slides untreated and treated with Corega.

Sample Condition	Mean OD (540 nm)			
	C	CTC	D	DTC
**Untreated**	0.3881	0.4994	0.6146	0.5109
**Treated**	0.2002	0.2276	0.3091	0.2961
**Sample Condition**	**Standard Deviation**			
	**C**	**CTC**	**D**	**DTC**
**Untreated**	0.3198	0.3788	0.3653	0.3963
**Treated**	0.0903	0.1851	0.1873	0.1649

**Table 4:** Correlation (Pearson) between the amount of biofilm and roughness, on untreated and treated surfaces.

Correlation	Surface	Pearson Coefficient Value
amount of biofilm-Sa 2	untreated	0.59
amount of biofilm-Sa 4	untreated	0.64
amount of biofilm-Ra	untreated	0.53
amount of biofilm-Ra	treated	0.89
amount of biofilm-GS 2	untreated	0.64
amount of biofilm-GS 4	untreated	0.47

## Data Availability

The original contributions presented in the study are included in the article; further inquiries can be directed to the corresponding authors.
